# Encapsulins—Bacterial Protein Nanocompartments: Structure, Properties, and Application

**DOI:** 10.3390/biom10060966

**Published:** 2020-06-26

**Authors:** Anna N. Gabashvili, Nelly S. Chmelyuk, Maria V. Efremova, Julia A. Malinovskaya, Alevtina S. Semkina, Maxim A. Abakumov

**Affiliations:** 1 Laboratory “Biomedical Nanomaterials”, National University of Science and Technology “MISiS”, Leninskiy Prospect, 4, 119049 Moscow, Russia; gabashvili.anna@gmail.com (A.N.G.); nellichmelyuk@yandex.ru (N.S.C.); 2 Department of Medical Nanobiotechnoilogy, Pirogov Russian National Research Medical University, Ostrovityanova st, 1, 117997 Moscow, Russia; alevtina.semkina@gmail.com; 3 Department of Nuclear Medicine, TUM School of Medicine, Technical University of Munich, 81675 Munich, Germany; mariia.efremova@helmholtz-muenchen.de; 4 Institute of Biological and Medical Imaging and Institute of Developmental Genetics, Helmholtz Zentrum München, 85764 Neuherberg, Germany; 5 D. Mendeleev University of Chemical Technology of Russia, 125047 Moscow, Russia; j.malinowskaya@gmail.com

**Keywords:** encapsulin, nanocompartment, cell labeling, MRI

## Abstract

Recently, a new class of prokaryotic compartments, collectively called encapsulins or protein nanocompartments, has been discovered. The shell proteins of these structures self-organize to form icosahedral compartments with a diameter of 25–42 nm, while one or more cargo proteins with various functions can be encapsulated in the nanocompartment. Non-native cargo proteins can be loaded into nanocompartments and the surface of the shells can be further functionalized, which allows for developing targeted drug delivery systems or using encapsulins as contrast agents for magnetic resonance imaging. Since the genes encoding encapsulins can be integrated into the cell genome, encapsulins are attractive for investigation in various scientific fields, including biomedicine and nanotechnology.

## 1. Introduction

The history of nanocompartments begins in 1994, when encapsulins were initially discovered as a high-molecular-weight complex in the bacterial culture supernatant of *Brevibacterium linens*, exhibiting bacteriostatic activity against various strains of *Arthrobacter, Bacillus*, *Brevibacterium*, *Corynebacterium*, and *Listeria* [1]. However, other encapsulins do not demonstrate such an effect, and even in the case of encapsulins from *Brevibacterium linens* in another study by Sutter et al., this fact was not confirmed [2,3]. Subsequently, homologous protein compartments were identified in the *Mycobacterium tuberculosis* [4] and *Thermotoga maritima* [5] culture supernatants. Moreover, these structures appeared to contain proteolytic enzymes. Encapsulins have also been identified during various studies in the bacteria *Mycobacterium leprae*, *Streptomyces*, and (much later) *Quasibacillus thermotolerans* [6,7,8,9,10,11]. In the mid-2000s, the observed high-molecular-weight aggregates were found to be protein capsid-like complexes [2,12,13].

The discovery of these structures spurred new research into prokaryotic nanocompartments. Some studies focused on the use of encapsulins as programmable nanoreactors or targeted delivery systems [14,15,16,17,18,19,20,21], while others investigated the physiological role of encapsulins and their cargo proteins in the native bacterial context. Bioinformatic analysis of sequenced genomes has revealed thousands of nanocompartment systems in both bacteria and archaea, with a wide variety of cargo proteins [20,22,23,24,25].

## 2. Structural Organization of the Shell

**Figure 1:**
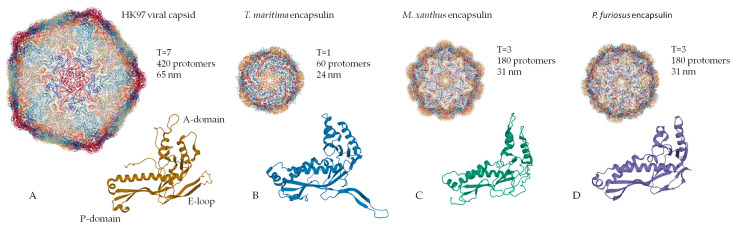
Structural comparison of the capsomers/monomers and assembled capsid/encapsulins: (**A**) HK97 phage, (**B**) *Thermotoga maritima* encapsulin, (**C**) *Myxococcus xanthus* encapsulin, (**D**) *Pyrococcus furiosus* encapsulin. (PDB-ID: 2FT1, 3DKT, 4PT2, and 2E0Z, respectively).

Encapsulin shells are icosahedral (12 vertices, 20 faces, 30 edges) complexes formed by self-assembly of protomer proteins [2,13,24]. Encapsulin shell proteins are homologous to gp5-HK97 phage main capsid protein (Figure 1A), as evidenced by the structural similarity between the gp5 protomer protein and encapsulin shell protein [26]. It should be noted that the HK97 fold is widespread in nature and has been observed in all other tailed phages [27], in herpesvirus capsids [28], and in the archaevirus HSTV-1 [29]. Like gp5, the encapsulin shell proteins oligomerize to form a complete nanocompartment. As well as viral capsids, encapsulin shell proteins can self-assemble into icosahedrons of different sizes. For example, encapsulins from *Pyrococcus furiosus* and *Myxococcus xanthus* consist of 180 protomers (30‒32 nm in diameter) (Figure 1C,D), while those from *Thermotoga maritima* are composed of 60 protomers (24 nm in diameter) (Figure 1B) and encapsulins from *Quasibacillus thermotolerans* consist of 240 protomers (42 nm in diameter) [2]. To classify this structural organization, a triangulation number can be used. The triangulation number (T) is a virology term describing the icosahedral packaging of viral capsid structural elements and is the quotient of dividing the number of subunits in a capsid by 60 [30]. Additionally, T can be explained as the number of subdivisions of each triangular facet of icosahedrons into identical equilateral triangles. The number of these triangles is equal to T. Following this classification, the *Pyrococcus furiosus* and *Myxococcus xanthus* encapsulins form icosahedrons with T equal to 3, *Quasibacillus thermotolerans* forms T = 4 icosahedrons, and *Thermotoga maritima* forms T = 1 icosahedron. The structure of an encapsulin shell protomer protein, like HK97 phage gp5 protomer, has three conserved domains: a peripheral domain (P), an axial domain (A), and an elongated loop (E) [2,6,14,30,31]. While the *Pyrococcus furiosus* and *Myxococcus xanthus* encapsulin protomer structures match the HK97 gp5 protomer structure well [13,24], only the A and E domains from the *Thermotoga maritima* encapsulin align well with those of the *Pyrococcus furiosus* and *Myxococcus xanthus* encapsulin protomer structures [2]. In particular, the E loop from *Thermotoga maritima* is shorter and rotated relative to the E loops of HK97 phage, *Pyrococcus furiosus*, and *Myxococcus xanthus* [2]. This allows the E loop to form a beta sheet with the E loop of a neighboring protomer, creating a tight interaction [2]. This may explain why the *Thermotoga maritima* encapsulin forms a T = 1 capsid, while the *Pyrococcus furiosus* and *Myxococcus xanthus* nanocompartments can form a larger T = 3 capsid.

It has been proposed that encapsulins and certain phage capsids have a common evolutionary origin [2,32]. This hypothesis is supported by the identification of genes encoding phage-like proteins close to the encapsulin gene in the archaeon *Sulfolobus solfataricus* [33].

Nanocompartments are also known to have multiple pores formed at the protomer junctions; the pore diameter is about 5 Å [2,13]. These holes are likely to serve as a permeability barrier for larger molecules while allowing small molecules and ions to pass across the shell. There is evidence that substrates of encapsulated enzymes, such as hydrogen peroxide or ferrous iron, can cross the shell, while proteins or other large molecules, such as DNA or polysaccharides, are not able to pass [17,18,22,25]. Interestingly, Williams et al. achieved an enlargement of pore diameter in the *Thermotoga maritima* encapsulin shell up to 11 Å using site-directed mutagenesis [34].

## 3. Cargo Proteins

**Figure 2:**
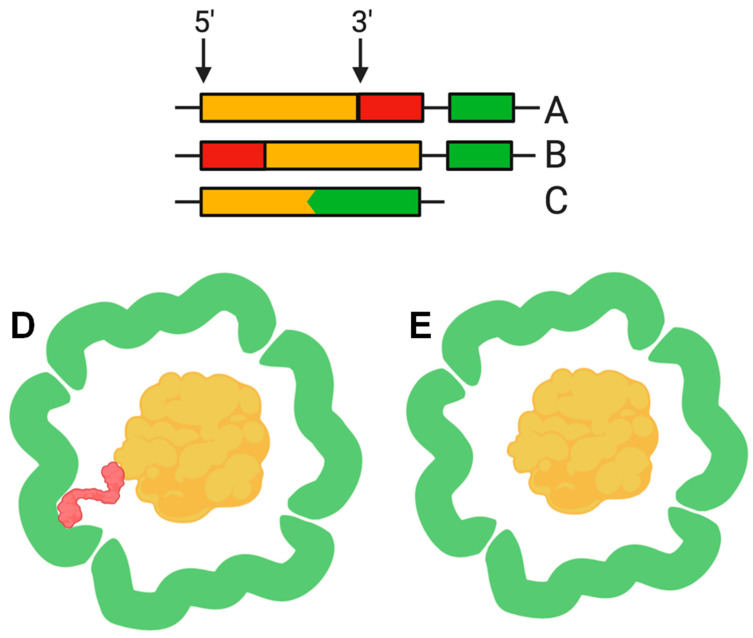
The cargo loading peptide coding sequence can be located at either the 5′ or 3′ end of the cargo loading peptide gene (**A**,**B**) with formation of the encapsulin structure (**D**). In some cases, the cargo protein encoding gene and encapsulin shell encoding gene are fused (**C**), and there is no need for a cargo-loading peptide to form encapsulin structure (**E**).

The function of the nanocompartment is associated with the function of its protein cargo. The first insight into the mechanism of cargo encapsulation was obtained when the crystal structure of the *Thermotoga maritima* encapsulin was investigated in detail. X-ray crystallography revealed a small amount of extra electron density related to a hydrophobic pocket on the luminal surface of the encapsulin shell corresponding to a short (about 10 amino acid) C-terminal sequence of a ferritin-like protein (FLP), found next to the encapsulin shell gene in the *Thermotoga maritima* genome [2]. Bioinformatic analysis revealed that this C-terminal sequence is conserved in different species where the cargo protein genes and encapsulin shell genes are located together in a putative operon. Examples of such “predicted” cargo proteins include: FLP, DyP (dye-decolorizing peroxidase), hemerythrin, and rubrerythrin [2,20]. This C-terminal sequence, hereinafter termed the cargo loading peptide (CLP), is demonstrated to be sufficient for the loading of a cargo protein into the nanocompartment shell. The CLP coding sequence can be located at the 3′ or 5′ end of the cargo protein encoding gene (Figure 2A,B,D). Deletion of CLP from the cargo protein disrupts encapsulation, while fusion of the CLP with the C-terminus of heterologous proteins, such as green fluorescent protein or luciferase, is enough for loading [2,15,17,18,20,23]. Nevertheless, there are alternative models describing the interaction between shell proteins and cargo proteins. In some cases, such as the *Pyrococcus furiosus* encapsulins, CLP is absent but the gene encoding the shell is fused with the gene encoding the cargo protein, forming a single polypeptide (Figure 2C,E) [13]. In the encapsulins found in *Firmicute* bacteria, loading may occur partially via an N-terminus of CLP. Such a nanocompartment contains both an iron-mineralizing encapsulin-associated firmicute protein (IMEF) with C-terminal CLP and a ferredoxin protein with an N-terminal CLP [6].

There is evidence that several cargo proteins can be loaded into a single encapsulin shell. For example, three different cargo proteins (EncB, EncC, and EncD) were detected in *Myxococcus xanthus* encapsulins [24], while in *Mycobacterium tuberculosis* the following proteins were found: Mt-DyP, Mt-BfrB (bacterioferritin), and Mt-FolB (folate biosynthesis enzyme) [23]. Another important issue related to loading is the fraction of the cargo protein within the nanocompartment. Each shell protein in encapsulin has a CLP binding site, but the amount of cargo is obviously limited by the shell volume. Modeling studies have shown that it is impossible to achieve a cargo-to-protein protomer ratio of more than 1:1 due to steric hindrance [2]. An example is the *Brevibacterium linens* nanocompartment with the mentioned DyP enzyme as a cargo protein that assembles into a hexamer (trimer of dimers) 89 Å in diameter. Steric hindrances were predicted to limit the loading to one hexamer per nanocompartment [2], and measurements by native mass spectrometry confirmed the presence of six DyP monomers in the T = 1 nanocompartment, resulting in a cargo-to-protein protomer ratio of 1:10 [35].

## 4. Stability as the Main Property of Encapsulins

Talking about the functions of encapsulins, it is worth mentioning that the biological purpose of the cargo protein encapsulation in most organisms remains unknown, since there are homologous forms of cargo proteins that are not encapsulated. For example, it is assumed that the encapsulation is not required for the enzyme function of a cargo protein such as DyP [2]. Some observations, however, support the hypothesis that encapsulation may increase the stability and/or lifetime of cargo proteins, for instance by increasing resistance to proteases and other factors. *Thermotoga maritima* encapsulins, for example, are extremely resistant to high temperature and denaturation [2,18], and *Brevibacterium linens* encapsulins are stable over an extremely wide pH range [36]. Like viral phage capsids, nanocompartments exhibit minimal degradation following treatment with nonspecific proteases [17,18]. Resistance to proteases also extends to the cargo proteins: firefly luciferase packaged into *Rhodococcus erythropolis* N771 encapsulins showed no degradation after trypsin treatment, while nonencapsulated luciferase completely degraded [17]. Encapsulins are often found in the bacterial culture supernatants [1,22,37], which allows us to hypothesize that nanocompartments are the product of bacterial secretion. This was confirmed by the observation of encapsulin localization on cell membranes [38]. However, such localization is not found in all prokaryotes; for example, *Streptomyces griseus* nanocompartments are located in the cytoplasm [11]. In addition, the secretion mechanism for an intact 25–35 nm protein complex is unknown [37]. Considering the extremely high chemical stability and resistance to proteases, an alternative version in which encapsulins accumulate in culture supernatants due to lysis after cell death in media was suggested [38].

## 5. The Physiological Role

Not much is known about the role of encapsulins in the metabolism of bacteria and archaea. To date, the most informative results come from studies of encapsulins containing ferritin-like proteins. The data suggest that nanocompartments can deposit iron, mitigating oxidative stress. A striking example is the bacterium *Quasibacillus thermotolerans*, which encodes no ferritins in its genome [31]. In another study, when *Myxococcus xanthus* cells were subjected to amino acid starvation, McHugh et al. found that the expression of the encapsulin shell protomer (EncA) and its three cargo proteins FLP (EncB, EncC, and EncD) was significantly increased [24]. The authors hypothesized that encapsulins can act as a secondary ferritin-like system that has a larger capacity and is able to accumulate iron during a forced starvation stress or sequester iron during oxidative stress. When exposed to reactive oxygen species, ferrous iron (Fe^2+^) is oxidized to ferric iron (Fe^3+^) via the Fenton reaction, yielding the formation of a hydroxyl radical as a by-product [39,40], while ferritins protect the cells against the toxic effects of this product. To test whether encapsulins could similarly protect a cell from oxidative stress, the authors evaluated the ability of a *Myxococcus xanthus* mutant strain lacking the sequence encoding the encapsulin shell to survive the oxidative stress induced by hydrogen peroxide, finding that the mutant was significantly more sensitive than the wild-type strain [24]. Encapsulins in *Mycobacterium tuberculosis* have the same property [23]: each of the three previously mentioned cargo proteins (BfrB, FolB, and DyP) has antioxidant activity [41,42,43,44].

In addition to their potential role in mitigating oxidative stress, DyP-containing encapsulins are also implicated in catabolism [22]. For example, a mutant strain of the bacterium *Rhodococcus jostii* RHA1 with the deletion of DyP encoding gene is incapable of lignin degradation [45], while lignin catabolism is active in the wild-type strain. Some studies have also shown that the nitrated-lignin-degrading activity of the encapsulin-DypB complex increased 8-fold compared to the nonencapsulated DypB enzyme [22]. An increase in enzymatic activity upon encapsulation suggests that the nanocompartment can act either by stabilizing the cargo protein or by increasing the local substrate concentration for the enzyme, thereby enhancing the enzymatic reaction [46].

## 6. Nanocompartments and Nanotechnology

Over the last decade, a variety of nanoscale targeted delivery systems based on micelles [47,48], liposomes [49], inorganic [50] and polymer [51] nanoparticles, and protein compartments [52,53] have been developed. The small size and easy surface functionalization of these particles can increase the efficiency of drug delivery and accumulation compared with conventional nonencapsulated drugs. Such drugs are characterized by improved tissue penetration, longer circulation in the bloodstream, and reduced side effects [54]. Additionally, targeted drug delivery systems can play a significant role in the diagnosis of diseases by interacting with specific molecular markers expressed in a particular pathology. However, none of the existing nanoscale systems (liposomes, inorganic and polymer nanoparticles) can be encoded into the genome and self-assembled by the cell, whereas the use of encapsulins opens up prospects for this particular application. Encapsulins are mainly used as a diagnostic tool or as a drug delivery system.

In various studies, *Thermotoga maritima* (Encap) encapsulins were used as a delivery system for both fluorescent probe and therapeutic drug when the nanocompartments were functionalized to bind to SP94, a hepatocellular carcinoma (HepG2) marker [14,55,56]. The Encap shell protein contains two cysteine residues (C123 and C197), while C123 is located on the outer surface of Encap, allowing for SP94 peptide conjugation to the encapsulin surface. Then, a fluorescent probe fluorescein-5-maleimide was added to the shell surface. Thus, the obtained nanodevice was able to specifically bind to cells and visualize SP94 simultaneously due to the presence of a fluorophore. It was further demonstrated that a prodrug aldoxorubicin ((6-maleimidocaproyl) doxorubicin hydrazone) could be loaded into the designed construct and released under acidic conditions inside tumor cells. The dose-dependent cytotoxicity of the drug against HepG2 cells was also demonstrated.

Encapsulins can be successfully modified with photo-switchable fluorophores [57]. The *Brevibacterium linens* encapsulin was functionalized with spiropyran-based fluorophores (organic compounds with photochromic properties) via the carbodiimide method [58]. Upon irradiation with ultraviolet and visible light, spiropyrans are able to switch between their fluorescent merocyanine photo-isomer and nonradiative isomer, allowing them to “turn on” and “turn off” fluorescence [58]. The authors demonstrated that modified encapsulins carrying spiropyran molecules on their shell remained stable through at least five cycles of photo-switching.

Modification of encapsulins with fluorescent dyes allows for investigating their intracellular fate and in vivo distribution, enabling a further understanding of their biological role and function. The possibility to switch the fluorescence on and off can significantly increase imaging resolution when using such super-resolution microscopy techniques as stochastic optical reconstruction microscopy (STORM) and photoactivated localization microscopy (PALM).

In particular, spiropyran derivatives are widely used in live-cell imaging [59]. The sufficient brightness and versatile chemical modification of spiropyran-based compounds allow the attachment of multiple fluorophore molecules to the encapsulin shell [60]. The high sensitivity of spiropyrans to redox changes as well as to changes in temperature and pH make these molecular switchers an emerging tool in the development of fluorescent biosensors [61]. Moreover, due to their biocompatibility and ability to penetrate cells, encapsulin-based fluorescent probes may also facilitate cell labeling and tracking. Simultaneous encapsulin modification with fluorescent labels and targeting moieties might also lead to the creation of an encapsulin-based targeted imaging probe. In another study [36], *Brevibacterium linens* encapsulins loaded with teal fluorescent protein (TFP) were found to be successfully captured by J774 murine macrophages in cell culture in vitro, yielding the intense fluorescence of macrophages. In this case, encapsulins remain within the cytoplasm without entering the nucleus. The authors note that the selected fluorescent model cargo can be replaced by a therapeutic agent and an encapsulin-based platform can be used for its delivery.

It was also shown that *Thermotoga maritima* encapsulins could be genetically modified to express the FcBP peptide (IgG-Fc domain-binding peptide) [62], which exhibits a high affinity to the rabbit IgG-Fc fragment [63]. Indeed, real-time quartz crystal microbalance (QCM) and surface plasmon resonance (SPR) analysis indicated that FcBP is displayed on the outer surface of encapsulin and is available for binding by rabbit IgG-Fc.

While speaking about targeted delivery systems, it is worth mentioning that the encapsulin shell surface of *Rhodococcus erythropolis* N771, described above, can be successfully coated with polyethylene glycol (PEG). PEG is well known as a biocompatible modifier of drug carriers, making them undetectable by the cells of the monocytic macrophage system (mononuclear phagocyte system) and reducing aggregation. The authors also showed that PEGylation does not affect the self-assembly of the nanocompartment [21].

In one study, *Thermotoga maritima* encapsulins were used as carriers of the influenza A virus protein M2 ectodomain (M2e epitope) [64]. The M2 protein forms ion channels on the virion surface and is essential for the transport of viral ribonucleoprotein complexes into the cytoplasm of the host cell. The amino acid sequence of the M2 protein is highly conservative, and its immunogenicity during natural infection is quite low [65]. These properties make M2e a good broad-spectrum vaccine candidate. In this work, anti-M2e epitope specific antibodies were determined in mouse serum following immunization with the obtained constructs.

Due to their size, protein compartments excellently mimic the defined intracellular environment and allow for studying the enzyme kinetics in more natural conditions [66,67]. It was shown that non-native proteins such as GFP and firefly luciferase (Luc) could be loaded into the encapsulin shell of *Rhodococcus erythropolis* [17]. Moreover, GFP retains its fluorescence properties, and luciferase showed enzymatic activity towards its substrate, luciferin. A similar study was conducted with *Brevibacterium linens* encapsulins. The C-terminal sequence of the native DyP cargo protein was fused to the C-terminus of TFP [68]. After confirming the structural integrity of the isolated nanocompartments, one encapsulin was found to contain 12 TFP molecules on average.

Interestingly, the encapsulins can be disassembled into protein subunits at acidic pH, loaded with a cargo of interest due to the affinity to certain protein sequences of the membrane, and finally reassembled under neutral pH conditions. Thus, molecules/particles with a size exceeding the nanocompartment pore diameter can be loaded into the encapsulin cavity. Thus, the authors of [69] chose gold nanoparticles with a diameter of 13 ± 1 nm, coated with (11-mercaptoundecyl)-N,N,N-trimethylammonium bromide, as cargo. These nanoparticles were loaded into the *Thermotoga maritima* encapsulin cavity by partially replacing the nanoparticle-stabilizing ligand with CLP.

This approach could be potentially applied for photothermal therapy of tumors, requiring the introduction of stable and biocompatible gold nanoparticles into the region of interest, followed by irradiation with the required wavelength and subsequent death of tumor cells, which are more sensitive to temperature increase compared to healthy cells.

## 7. Nanocompartments as Cell and Tissue Markers

To date, the main possible approaches to the problem of cell monitoring once they are introduced into the body are (1) direct labeling methods, where exogenous labels are added to the cell culture medium and bind to cells in ex vivo conditions (magnetic nanoparticles, radioactive isotopes, and low-molecular-weight fluorophores [70,71]) and (2) reporter genes—the endogenous labels expressed by cells as a result of the corresponding gene introduction at the cell cultivation stage (luciferase, fluorescent proteins, etc. [72]). However, the fluorescent imaging is limited to a penetration depth of about 2 mm; therefore, various quantum dots and fluorophores can be applied only for surface tissue imaging in small animals, e.g., mice [73,74]. One promising approach is the search for genetically encoded reporters for the visualization technique that allows for noninvasive whole-body imaging, such as MRI.

Magnetic resonance reporter genes (MRI reporter genes) produce intracellular metalloproteins, most commonly transferrin, ferritin, or tyrosinase [75]. It is well known that MRI can detect iron atoms due to their paramagnetic properties. Transferrin (TfR) contains two iron atoms, allowing their intracellular accumulation, so in this case the main task is to increase the expression of this protein. Ferritin may contain up to 4000 iron atoms, but it does not possess pronounced magnetic properties, since it mostly comprises antiferromagnetic ferrihydrite [76]. To improve the T2 relaxivity of ferritin, its native ferrihydrite core can be removed and replaced with a superparamagnetic core. Thus, the authors of [77] inserted a reporter ferritin gene into mouse skeletal myoblasts, followed by transplantation to a mouse heart damaged by a heart attack. As a result, excessive ferritin expression did not alter cell viability, but caused a significant increase in T2 relaxivity. Another metalloprotein tyrosinase is responsible for melanin production, which in turn has high iron affinity, contributing to an increase in the MRI signal. Fibroblasts, embryonic kidney cells, and breast cancer cells were successfully transfected with the gene of this protein [78].

Another promising approach is based on encapsulin-based reporters, which can be expressed in mammalian cells using bioengineering [79,80]. Thus, encapsulins allow iron sequestration via the formation of 10–30-nm particles, which could possibly enable the MRI imaging of the cells.

In one study [79], *Quasibacillus thermotolerans* (Qt) and *Myxococcus xanthus* (Mx) encapsulin expression was achieved by transient co-transfection in HEK293T cells. The cells were transfected with plasmid DNA encoding the encapsulin shell, its cargo protein (ferroxidase enzyme), and iron transporter, which is required for more efficient iron transport. Twenty-four hours post-transfection, ferrous ammonium sulfate, as a source of ferrous iron oxidized by ferroxidase, was added to the cells.

Cryo-electron microscopy revealed that the encapsulin Qt shell self-assembles into nanocompartments with T = 4 icosahedral symmetry and a diameter of ~43 nm. Additionally, the native cargo protein was found to retain its ferroxidase activity, allowing effective iron biomineralization. In particular, the Mx ferritin-like cargo protein was shown to mineralize up to 30,000 iron atoms per nanocompartment, which is approximately an order of magnitude more than the amount ferritins can deposit.

Another study [80] demonstrated that two cargo proteins, namely, fluorescent PAmCherry and ferritin-like protein B, can be simultaneously loaded into *Myxococcus xanthus* encapsulins. The authors also showed that HEK293T cells expressing encapsulins are able to store enough iron to be successfully separated on commercial magnetic sorting columns (around 5% of the initial cell population) and can even be detected in vivo via MRI upon administration to the rat brain.

Heterologous expression of encapsulins can be achieved with high probability, not only in HEK293T cells, but also in other cell lines or mammalian cell cultures. Importantly, compared to traditional medicines based on small molecules or nanoparticles, cell-based therapeutics are much more complicated systems, requiring careful monitoring to avoid undesired differentiation, cell migration, or proliferation. Thus, such a therapeutic approach requires noninvasive monitoring of the cells upon administration to the patient.

Previous studies involved a transient transfection of HEK293T cells, allowing the transgene to be introduced into the cell genome only for a limited period of time, or special clone selection being applied to obtain a stable cell line expressing encapsulins. However, if stable integration of genes encoding encapsulins in the genome of a mammalian cell is achieved, protein expression will be observed in the daughter cells, resulting in a stable MRI signal.

## 8. Conclusions

Although the study of encapsulins began not so long ago, these unique structures have already been applied in various scientific fields. Properties of nanocompartments, such as the small size and possibility of surface functionalization for selective interaction with specific proteins, allow for the creation of various systems for targeted drug/label delivery. Encapsulins can be used as nanoreactors, allowing the investigation of enzyme kinetics in vivo. In addition, given the natural ability of certain nanocompartments to store iron, they can be employed as contrast agents for MRI to monitor cell distribution in the body, as described above. The discovery of new types of encapsulins in different bacterial strains, along with further investigation of these structures and their properties, seems to be very promising for a wide range of biomedical applications.
